# 3-Dimensional topographic enamel surface changes after different debonding techniques for aligner attachments: a micro-CT study

**DOI:** 10.1007/s00784-025-06188-6

**Published:** 2025-01-25

**Authors:** Hilal Turkoglu, Ezgi Atik

**Affiliations:** Faculty of Dentistry, Department of Orthodontics , Hacettepe University, Sihhiye, Ankara, 06100 Turkey

**Keywords:** Clear aligner, Attachment, Debonding, Polishing, Roughness, Micro-CT, Attachment debonding plier

## Abstract

**Introduction:**

To evaluate topographic changes of enamel surface in 3-dimensional after different debonding methods of aligner attachments formed with 2 different composite resins.

**Methods:**

Vertical rectangular attachments were created on 88 premolar teeth and divided into two composite resin groups (Group 1:flowable, Group 2:packable) (*N* = 44). These were then divided into two subgroups (*N* = 22) using different debonding methods. In Group A, the attachments were firstly removed using an attachment debonding plier and then with white fiberglass. Following, the tooth surfaces were polished with blue fiberglass. In Group B, the excess attachment composite was removed with a 12-blade carbide bur, followed by a 24-blade carbide bur, and tooth surfaces were polished with Renew stone. The remaining composite volume was measured using Geomagic Control X software. Enamel surface roughness and morphological change were compared between the groups.

**Results:**

Residual composite resin volume did not show a statistically significant difference between composite resin groups (1–2 A). The enamel demineralization volume and area changes in Group 2 A were significantly higher than observed in 2B (*p* < 0.05). Roughness parameters Ra (T1-T0), Ra (T2-T0), Rq (T1-T0), and Sa (T1-T0) were significantly higher in Group 1B compared to Group 1 A (*p* < 0.05). Similarly, Ra (T1-T0), Sa (T1-T0), and Sq (T1-T0) parameters were significantly higher in Group 2B compared to Group 2 A (*p* < 0.05).

**Conclusions:**

Fiberglass with a debonding plier produced a smoother enamel surface compared to carbide burs, but caused significantly more enamel demineralization, as seen in micro-CT evaluations after polishing.

## Introduction

The fact that clear aligner treatments are more aesthetic and comfortable than traditional fixed appliances has increased the demand for this treatment method [1]. The attachments used in the clear aligner system are designed to apply force systems to the teeth [2]. In clinical practice, composite resins of different viscosities with different filler amounts continue to be used in attachment production according to the clinician’s preference [3, 4]. The procedure after the completion of orthodontic treatment with clear aligners involves removing the attachments from the tooth surface and the enamel surface must be returned to its pre-treatment state as much as possible.​ Composite residues remaining on the tooth surface after debonding can contribute to biofilm buildup, thereby increasing the susceptibility to demineralization and dental caries [5]. On the other hand, residual resin removal at the debonding stage can create scratches, cracks, and grooves, increasing enamel roughness and causing enamel damage [6].

There is no consensus in the literature regarding which method is the most efficient and safe for removing resin residues. A variety of instruments and techniques, including ultrasonic cleaning, electrothermal cleaning, aluminum oxide rubber and sandblasting, discs, diamond burs, stainless steel burs, white stone, lasers, and fiber-reinforced composite burs, have been introduced and studied [7–11]. One of the most preferred methods for removing resin remnants is the tungsten carbide bur, used either with a low-speed or high-speed handpiece [9, 12]. As the rotation of these burs generates high force between the blades and the resin surface, it could lead to plastic plowing of the resin, as emphasized by Elliades et al. [13, 14]. Additionally, some researchers do not recommend tungsten carbide burs, as they may cause excessive enamel loss and increased surface roughness compared to other techniques [9, 11, 15, 16]. A new composite bur, enriched with zircon-reinforced glass fiber from Morelli Company, has gained popularity and is indicated for the safe removal of adhesive remnants and polishing the enamel for later finishing without damage, according to the manufacturer [17].

One essential step after debonding is polishing the enamel surface. Polishing minimizes the roughness of the enamel, and systems such as OneGloss, Sof-Lex, fiberglass, DU10CA-Ortho, Enhance finishing, PoGo polishing kit, and Renew polishing can be utilized for this purpose [6, 18–20].

While there are many methods to assess enamel damage, including scanning electron microscopy (SEM), stereo microscopy, and atomic force microscopy (AFM) [7], micro-CT stands out as an effective imaging technique that enables structural analysis at the micron level and provides 3-dimensional (3D) analysis [21].

To our knowledge, there is no study in the literature regarding debonding and polishing methods of the attachments created with composites in orthodontic treatments performed with clear aligners. This in-vitro study aimed to evaluate enamel surface roughness and topographic changes in 3D (with non-contact profilometer and micro-CT) after the removal of aligner attachments formed with two different composite resins which were flowable (GC Universal Injectable) and packable (GC Genial Restorative) using two different debonding methods (attachment debonding plier followed by fiberglass bur application and tungsten carbide bur followed by Renew stone application). Additionally, it was aimed to calculate the remaining composite volume after removing different composite resins from the tooth surfaces in the groups where the Plier method was applied. The null hypothesis tested in this study was that there was no difference in enamel surface roughness and topographic changes after removing aligner attachments created with two different composite resins using different debonding methods. In addition, it was assumed that there was no difference in the remaining composite volume after removing different composite resins from the tooth surfaces in the groups where the Plier method was applied.

## Materials and methods

### Sample selection and preparing

The present in-vitro study was approved by Hacettepe University Non-Interventional Clinical Research Ethics Committee (approval number: GO 23/487). Assuming the significant difference required between the groups related to roughness measurement is 0.33 μm, taking into account the study conducted by Shah et al. [6], the required number of samples for each main group was determined as 44 with 80% power at a significance level of 0.05.

**Fig. 1:**
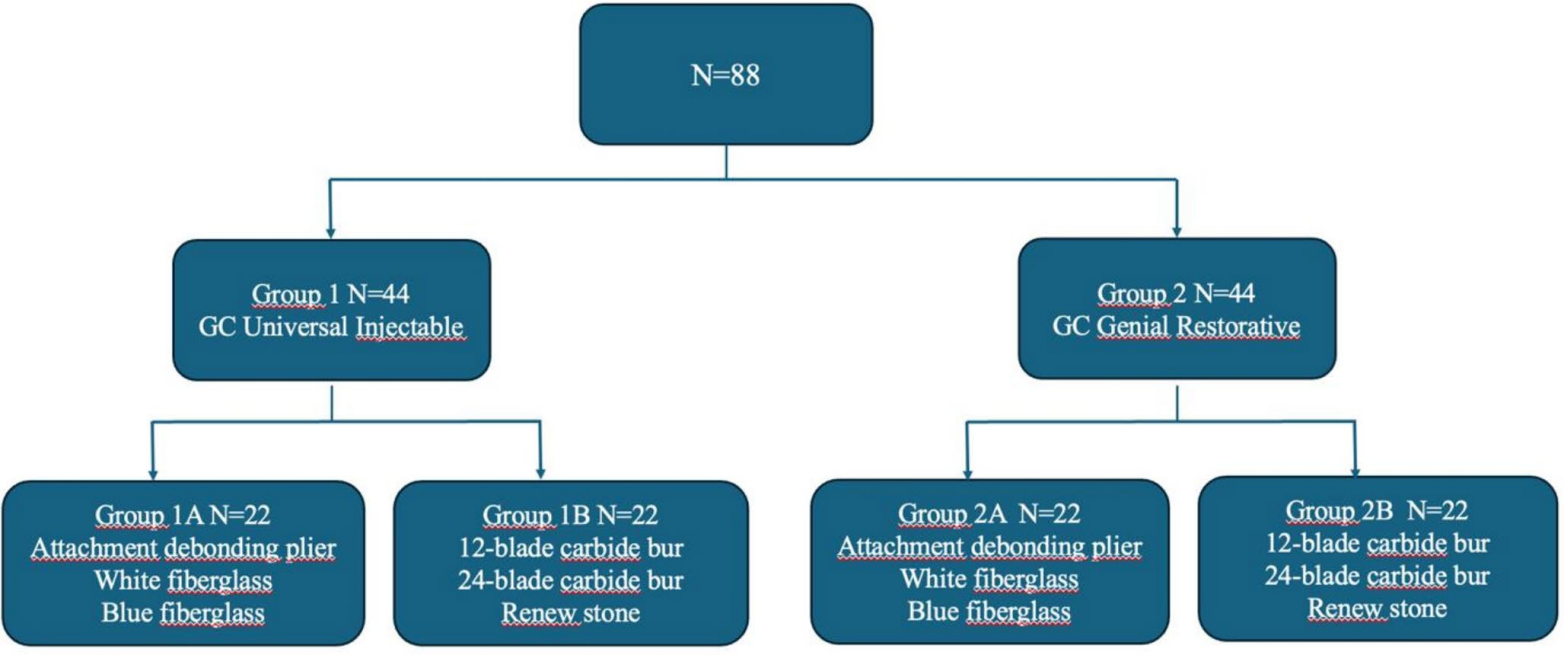
Study sample chart

Eighty-eight premolars with intact buccal surfaces, which were extracted for orthodontic purposes, were included. Teeth with caries, fractures, coronal cracks, white-spot lesions, enamel hypoplasia, and restorations were excluded. The samples were cleaned using fluoride-free pumice and rubber polishing for 10 seconds (s), followed by drying with an air spray. Subsequently, the teeth were randomly divided into two main groups based on the type of composite used with 44 teeth in each group (*N* = 44) (Group 1: Flowable GC Universal Injectable and Group 2: Packable GC Genial Restorative). These groups were further subdivided into two subgroups according to the different debonding techniques (A: Debonding plier, white fiberglass, and blue fiberglass) (B:12-blade carbide bur, 24-blade carbide bur, and renew stone), forming a total of four groups (*N* = 22). The specimens were grouped as shown in Fig. 1.

### Bonding of attachments to the tooth surfaces

**Fig. 2:**
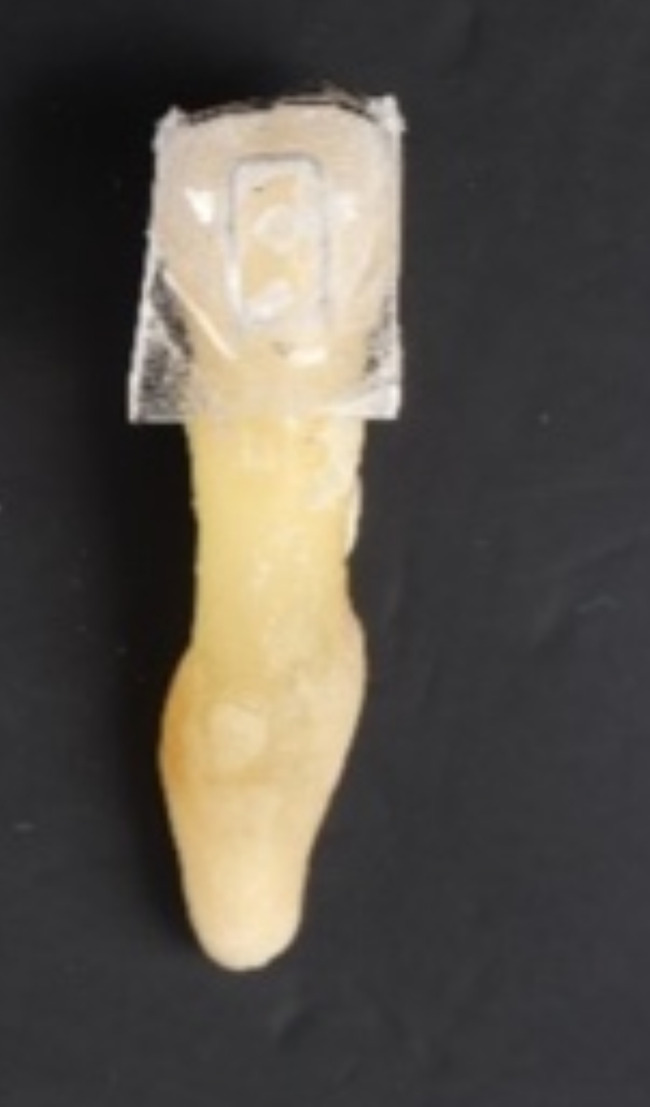
Attachment plate piece on which the roughness measurement areas on the enamel surface were determined

All teeth were embedded in plaster blocks with the crowns exposed and perpendicular to the horizontal plane (11 premolars forming a ‘U’ shaped dental arch) before the attachment plates were produced. The 8 study models were scanned with an iTero Element^®^ (iTero; Align Technologies, San Jose, Calif) digital scanner for attachment plate fabrication, and STL (stereolithography) images were obtained. Rectangular vertical attachments with dimensions of 4 mmx2 mmx1.25 mm were planned virtually by Orthero using Ortho Planner™ software for each premolar tooth. Two sets of template attachment plates were produced for each arch model. One of these templates was used for bonding the attachments to the tooth surfaces, and the other was used as a key plate to standardize the roughness measurement area on the enamel surface (Fig. 2). The attachment parts of the plates were cut off with utmost precision, exposing the measurement areas. The measurements were taken before attachment bonding (T0), after the debonding procedure (T1), and after polishing (T2). The plate piece corresponding to each tooth was then attached to the tooth, and the area defined by the attachment region cut from the plate was measured. This procedure ensured that the same area was measured at all three different time points, maintaining the high standards of our research.

The teeth were roughened with 37% orthophosphoric acid for 30 s, then rinsed with water and air dried. G-Premio Bond (GC Corp, Tokyo, Japan) was applied and cured with light. In Group 1, GC Universal Injectable flowable (GC Corp, Tokyo, Japan) composite was injected into the attachment reservoir of the plate, and in Group 2, GC Genial Restorative packable (GC Corp, Tokyo, Japan) posterior composite was placed into the attachment reservoir using a mouth spatula. Composite polymerization was achieved with a light device (VALO Ortho; Ultradent Products, South Jordan, Utah) for 3 s at 3200mW/cm². Afterwards, all specimens were incubated in distilled water for 24 h to complete polymerization.

### Debonding and resin removal procedures

**Fig. 3:**
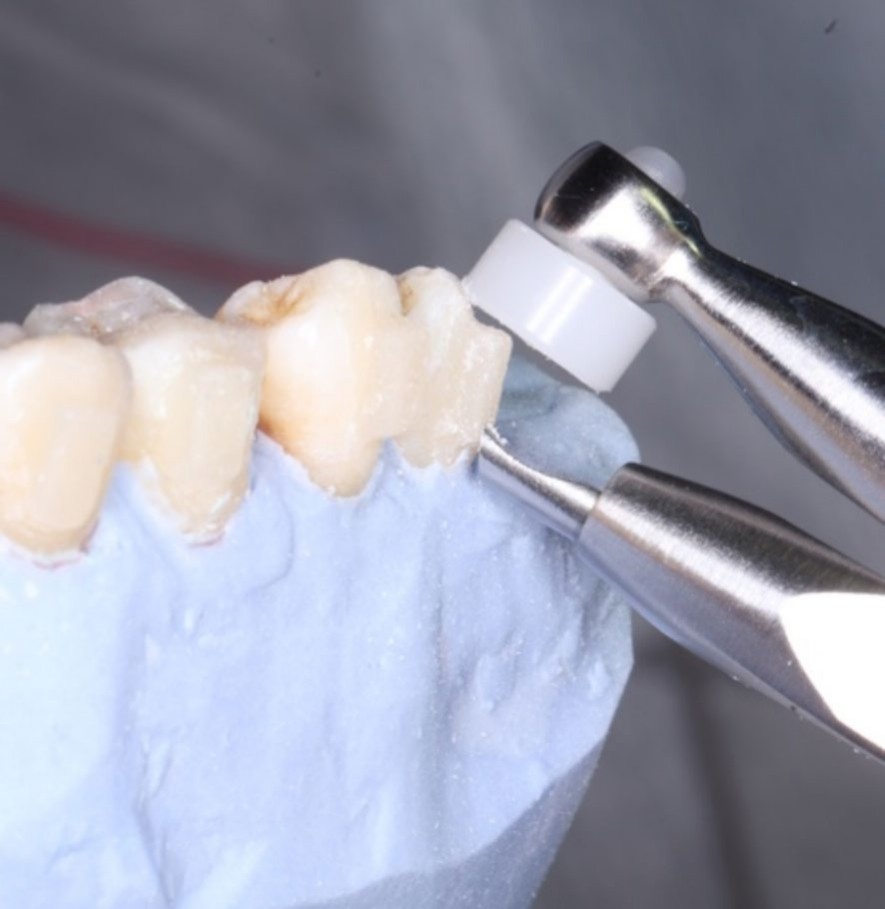
Removing the attachment from the tooth surface with the attachment debonding plier

**Fig. 4:**
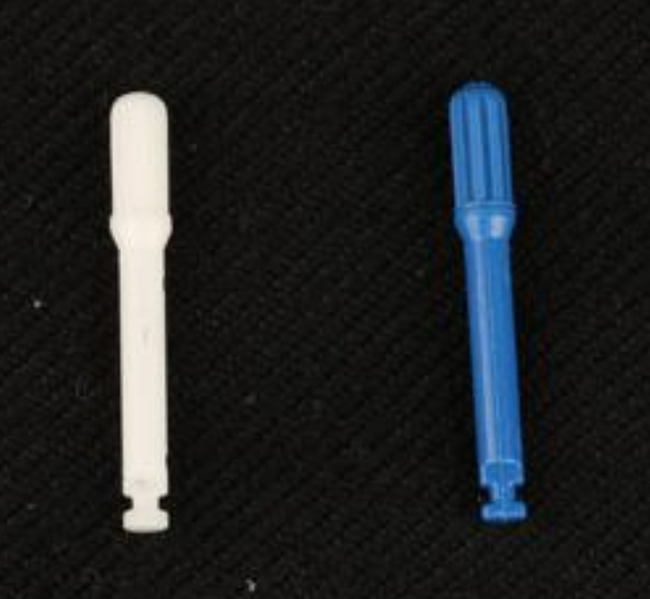
White and blue fiberglass

For the teeth in Groups 1 A and 2 A (*N* = 22), the attachments were removed from the tooth surfaces using an attachment debonding plier (Fig. 3). Then, the composite resins remaining on the tooth surfaces were removed with “white fiberglass” (Morelli, Brazil) using a low-speed handpiece of 20.000 rpm. This process was followed by polishing the tooth surfaces with “blue fiberglass” (Morelli, Brazil) (Fig. 4).

**Fig. 5:**
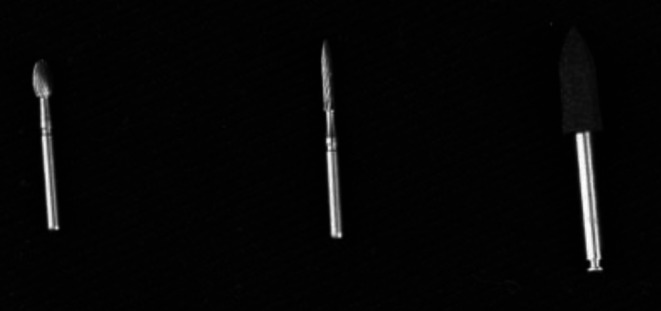
12–24 blade tungsten carbide bur- renew polishing stone

For the teeth in Groups 1B and 2B (*N* = 22), the thick excess composite attachments were initially removed using a 12-blade carbide bur (Frank Dental, Germany) with a high-speed handpiece. The remaining composite was then removed with a 24-blade carbide bur (Frank Dental, Germany) utilizing a low-speed handpiece at 20,000 rpm as the enamel surface approached. Following this process, Renew stone (Renew Finishing System; Reliance Orthodontic Products Inc., IL, USA) was used for polishing (Fig. 5). No water was applied during the debonding and polishing stages. A single operator (H.T) performed all bonding and debonding procedures. For each sample, a new bur and polishing stone were used to ensure cutting and polishing efficiency. Resin removal was considered complete when the enamel surface was smooth and free of composite to the naked eye under conventional lighting emitted by a dental chair unit.

**Fig. 6:**
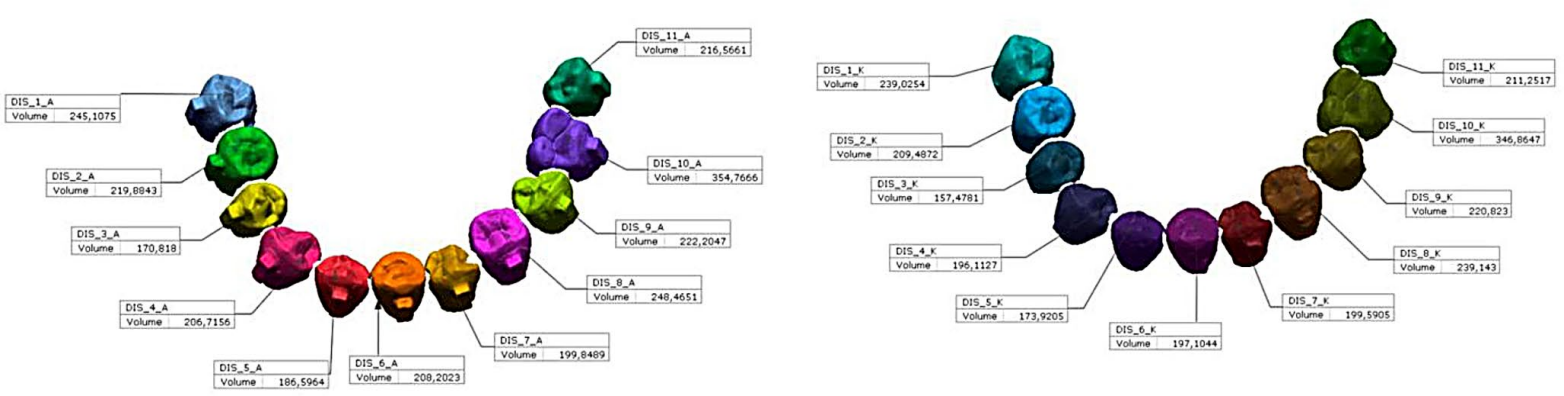
**A**: Volume values of the model with attachments applied, **B**: Volume values taken after removal of the attachments with debonding plier

For the groups that used the attachment debonding plier (Group 1–2 A), the remaining composite was quantitatively evaluated using Geomagic Control X (3DS Systems, Rock Hill, South Carolina, USA) software after removing composites of different viscosities from the tooth surfaces (Fig. 6). During the superimposition process of the attachments, the occlusal surfaces of the teeth served as reference points, and the ‘best-fit’ method was applied. Each tooth was segmented following the superimposition process. Separate volume measurements were taken for the teeth with attachments and the teeth after debonding the attachments using the software.

### Evaluation of enamel roughness with 3D optical profilometer

Measurements of two-dimensional and three-dimensional surface roughness, as well as area and line roughness values, were conducted on all teeth involved in the study (*N* = 88) prior to attachment bonding (T0), following debonding (T1) and after polishing (T2) using a 3D optical profilometer (Filmetrics Profilm 3D, KLA).

White light interferometry (WLI), a non-contact optical technique, was used to measure the surface profiles and roughness of the samples with the Profilm3B optical profilometer. The samples were scanned using 20X magnification lenses with a field of view of 1.0 × 0.85 mm within a 500 × 500 μm area, with spatial sampling at 2X zoom. The collected raw data were analyzed with Profilm software. The following roughness measurements were calculated:

Ra: The average roughness value, defined as the arithmetic mean of the height of peaks and the depths of valleys from a mean line, measured in nanometers.

Rq: Root mean square roughness refers to the height distribution in nanometers relative to the mean line.

Sa: The arithmetic average of the heights of the peaks and the depths of the troughs for the sampled area.

Sq: Height distribution by average area.

**Fig. 7:**
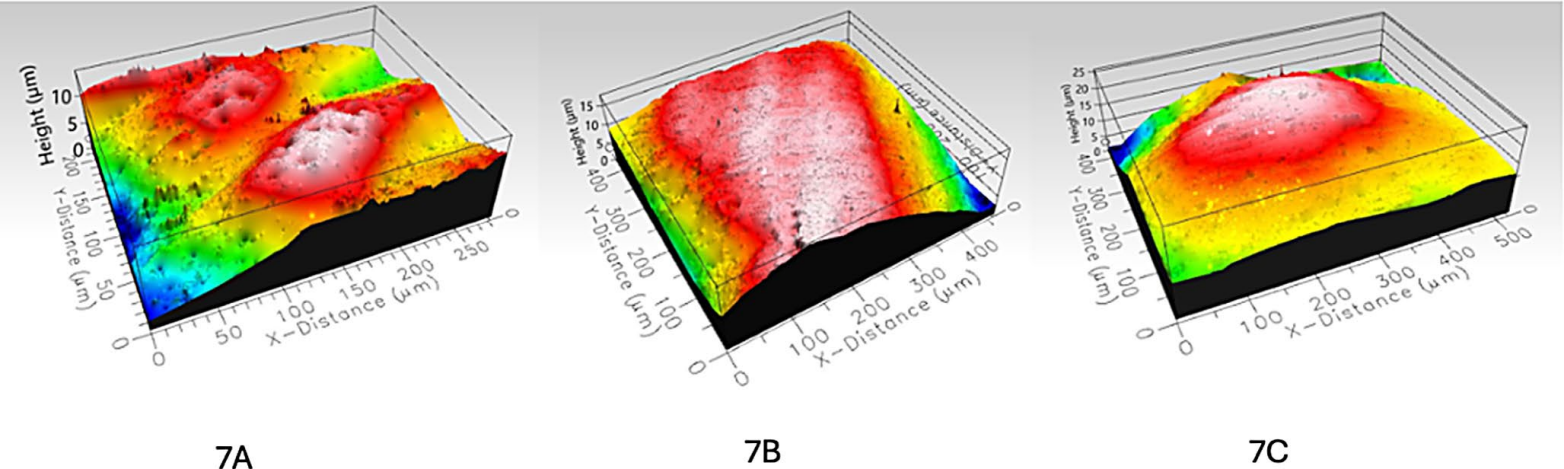
Enamel surface roughness measurement images of the sample tooth from Group 1 A taken by 3D optical profilometer at T0 (7 A), T1 (7B), and T2 (7 C) periods

Figure 7 shows the enamel surface roughness measurement images of the sample tooth from Group 1 A taken by 3D optical profilometer at baseline (T0), after debonding (T1), and after polishing (T2).

### Evaluation of enamel surfaces with micro-CT

For micro-CT evaluation, 15 teeth for each group were selected from Groups 2 A and 2B. Two measurements were performed on the selected teeth before attachment bonding (T0) and after polishing (T1). The amount and area of enamel tissue removed from the tooth surface were assessed using micro-CT application on selected teeth. Teeth were scanned using a high-resolution micro-CT system (Bruker Skyscan 1275, Kontich, Belgium) at 80 kVp, 125 mA, with an Al/Cu filter, a pixel size of 22 μm, and settings for 0.2 step rotation. The scanning time was approximately 1 h. Each sample was rotated 360° within a 5-minute integration time. Each tooth was scanned twice before and after the debonding procedure of the attachments to ensure standardization of the study with the same scan parameters.

**Fig. 8:**
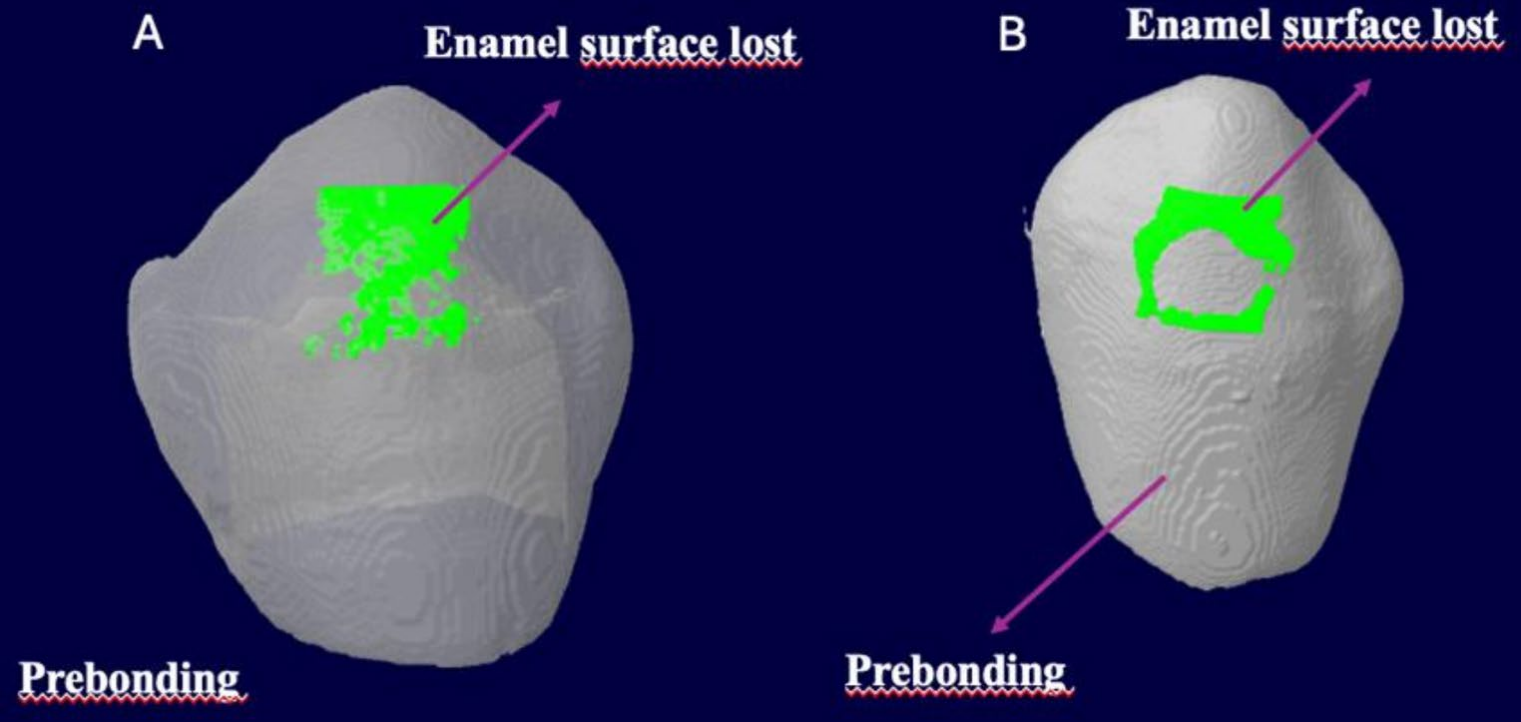
Example photographs of the subgroups showing the enamel surface before bonding (white) and the lost enamel surface (green) after debonding and polishing. A-Group 2 A (fiberglass application after debonding plier), B-Group 2B (renew stone after 12 and 24-blade tungsten carbide bur)

Imaging and quantitative measurements of the samples were performed using NRecon (ver. 1.6.10.4, SkyScan, Bruker, Billerica, MA, USA) and CT-analyzer software (CTAn, ver. 1.17.7.2, SkyScan, Bruker, Billerica, MA, USA), which provided axial, two-dimensional (2D), 1000 × 1000-pixel images. DataViewer software (version 1.5.6.2, SkyScan, Bruker, Billerica, MA, USA) was initially used to superimpose the reconstructed images. Then, the CTAn software (SkyScan, Bruker, Billerica, MA, USA) was used for 3D volumetric visualization and volume measurement. The calculated parameters included the demineralization area (mm^2^), demineralization depth (mm), and demineralization volume (mm^3^) following adhesive removal. An automatic segmentation threshold was also used to analyze the mineral density (g/cm³), processing the range of gray levels to produce a superimposed image of black and white pixels. Example scans of the subgroups indicating the enamel surface before bonding and after debonding and polishing are shown in Fig. 8.

### Statistical analysis

The data obtained from the study were analyzed using IBM SPSS Version 23 (Statistical Package for Social Sciences). The normality distribution was assessed using the Shapiro-Wilk test. The descriptive statistics presented in this study included the mean, standard deviation, median, minimum, and maximum.

For Geomagic Control X (adhesive removal amount) and Micro-CT measurements, the Independent Sample T-test was used to compare two independent groups with normal distribution, while the Mann-Whitney U test was applied when the assumptions were not satisfied. For micro-CT measurements, the difference between two dependent variables (time) within the group was evaluated using the Paired Sample T-test.

For roughness measurements, the difference between two dependent variables (time) within the group was evaluated using the Paired Sample T-test when the assumptions of the parametric test were satisfied, and the Wilcoxon test when they were not. The effects of the two factors of interest (Factor 1: Group 1 or 2; Factor 2: Group A or B) on the changes between times were evaluated using the Bonferroni pairwise comparison test results obtained from the two-way ANOVA analysis.

For roughness measurements, the statistical significance level was *p* < 0.017 for the changes within the group, whereas this level was *p* < 0.05 for all other analyses.

## Results

### Evaluation of the remaining attachment composite volume using Geomagic X software

The attachment volume removed, calculated using Geomagic software after plier use, is shown in Table 1. The median attachment volume change in Group 1 A was 7.72 (0.49–9.83) mm³, while in Group 2 A it was 7.40 (0.26-10) mm³. No statistically significant difference was found between the composite resin groups regarding the attachment volumes removed.

**Table 1:** Comparison of changes in attachment volume after removing attachments with debonding pliers

Composite Resin Groups	*N*	Median (Min.-Max.) (mm^3^)	Mean ± SD (mm^3^)	*p*- between groups
Group 1 A (GC Universal Injectable)	22	7.72 (0.49–9.83)	6.88 ± 2.58	0.851
Group 2 A (GC Genial Restorative)	22	7.40 (0.26-10)	6.99 ± 2.48	

Mann-Whitney U test was used

*p < 0.05 was significant

Min.=Minimum; Max.=Maximum; SD = Standard deviation

### Evaluation of topographic changes on the enamel surface with micro-CT

The volume and area of demineralization in Group 2 A were significantly greater than in Group 2B (*p* < 0.05). However, no statistically significant difference was observed between the groups regarding the median change in demineralization depth (Table 2).

**Table 2:** Comparison of topographic changes in enamel surface (volume, Area, and depth) obtained through micro-CT between different debonding methods in the packable posterior composite resin group

Variables	Groups	*N*	Mean ± SD	Median (Min.-Max.)	*p*
Demineralization volume (T1-T0) (mm^3^)	Grup 2 A	15	0.47 ± 0.11	0.49 (0.24–0.59)	0.006*
	Grup 2B	15	0.32 ± 0.16	0.30 (0.08–0.57)	
Demineralization area (T1-T0) (mm^2^)	Grup 2 A	15	0.55 ± 0.14	0.52 (0.21–0.73)	0.005*
	Grup 2B	15	0.30 ± 0.26	0.19 (0.07–0.86)	
Demineralization depth (T1-T0) (mm)	Grup 2 A	15	0.40 ± 0.12	0.39 (0.23–0.58)	0.056
	Grup 2B	15	0.30 ± 0.14	0.29 (0.15–0.59)	

Mann-Whitney U test was used

*p < 0.05 was significant

Min.=Minimum; Max.=Maximum; SD = Standard deviation

The evaluation of the groups regarding mineral densities on the enamel surface is shown in Table 3. For Groups 2 A and 2B, there was a statistically significant loss of mineral density following debonding and polishing (*p* < 0.05). No statistically significant difference was observed between the two groups regarding changes in mineral density (T1-T0).

**Table 3:** Comparison of enamel surface densities (g/cm³) obtained through micro-CT between different debonding methods (2 A-2B) in the packable posterior composite resin group and evaluation within the group

Groups	*N*	Measurement Time	Mean ± SD (g/cm^3^)	Median (Min.-Max.) (g/cm^3^)	*p*^b^- (T1-T0) Between groups
Group 2 A	15	T0	2.61 ± 0.09	2.62(2.47–2.78)	0.107
		T1	2.56 ± 0.09	2.56(2.41–2.74)	
		T1-T0	-0.05 ± 0.03	-0.05(-0.13_-0.01)	
		***Within group p*** ^***a***^ ***-(T1-T0)*** ***< 0***.001*			
Group 2B	15	T0	2.62 ± 0.07	2.63(2.49–2.75)	
		T1	2.55 ± 0.07	2.56(2.41–2.68)	
		T1-T0	-0.07 ± 0.04	-0.08(-0.14_-0.01)	
		***With in group p*** ^***a***^ ***-(T1-T0)*** **< 0**.001*			

*p < 0.05 was significant

p^a^: Paired Sample T-test was used

p^b^: Independent Sample T-test was used

Min.=Minimum; Max.=Maximum; SD = Standard deviation

### Evaluation of changes in enamel surface roughness using 3D optical profilometer

Data of enamel surface roughness measurements for each group is presented in Table 4. According to the intra-group evaluation, Groups 1 A, 1B, 2 A, and 2B showed a statistically significant increase in roughness values at T1 compared to the initial (T0) roughness value and a decrease at T2 when compared to the value at T1 (*p* < 0.05) (Table 5). Compared to time T0, a significant increase in Sa and Sq roughness values was observed in Groups 1 A and 1B at time T2. In contrast, there was no significant change in these parameters for Group 2 A; however, a significant increase in Sa was observed in Group 2B (Table 5).

**Table 4:** Data of enamel surface roughness measurements for T0, T1, and T2 periods of each group

		Group 1 A	Group 1B	Group 2 A	Group 2B
Variables	Time	Mean ± SD Median (Min.-Max.)	Mean ± SD Median (Min.-Max.)	Mean ± SD Median (Min.-Max.)	Mean ± SD Median (Min.-Max.)
Ra (µm)	T0	0.20 ± 0.12 0.15 (0.07–0.50)	0.15 ± 0.07 0.13 (0.07–0.31)	0.16 ± 0.07 0.13 (0.05–0.31)	0.14 ± 0.06 0.14 (0.05–0.34)
	T1	0.23 ± 0.10 0.21 (0.09–0.48)	0.25 ± 0.09 0.24 (0.13–0.46)	0.23 ± 0.07 0.22 (0.12–0.37)	0.27 ± 0.13 0.23 (0.1–0.56)
	T2	0.12 ± 0.04 0.11 (0.06–0.24)	0.17 ± 0.05 0.16 (0.08–0.26)	0.15 ± 0.04 0.14 (0.1–0.25)	0.14 ± 0.06 0.12 (0.05–0.29)
Rq (µm)	T0	0.26 ± 0.16 0.20 (0.10–0.66)	0.21 ± 0.09 0.18 (0.1–0.4)	0.21 ± 0.1 0.18 (0.07–0.42)	0.18 ± 0.08 0.19 (0.07–0.4)
	T1	0.29 ± 0.14 0.27 (0.12–0.67)	0.31 ± 0.09 0.29 (0.17–0.52)	0.29 ± 0.08 0.29 (0.16–0.49)	0.28 ± 0.08 0.27 (0,19 − 0,48)
	T2	0.22 ± 0.12 0.20 (0.08–0.52)	0.20 ± 0.07 0.19 (0.09–0.45)	0.20 ± 0.05 0.19 (0.12–0.33)	0.19 ± 0.08 0.19 (0.07–0.34)
	T0	1.49 ± 0.72 1.42 (0.65–3.64)	1.95 ± 0.54 1.99 (1.05–2.92)	2.61 ± 0.77 2.71 (1.01–3.92)	2.70 ± 0.8 2.67 (1.32–4.38)
Sa (µm)	T1	2.29 ± 0.61 2.20 (1.27–3.61)	3.39 ± 0.65 3.33 (2.37–4.74)	3.89 ± 0.99 3.90 (2.11–5.73)	4.70 ± 1.28 4.71 (1.21–7.52)
	T2	2.25 ± 0.76 2.20 (1.18–3.68)	3.21 ± 0.75 3.28 (1.47–4.99)	3.18 ± 0.78 3.16 (1.51–4.87)	3.52 ± 0.82 3.57 (2.02-5)
	T0	1.82 ± 0.88 1.69 (0.78–4.43)	2.43 ± 0.69 2.45 (1.26–3.9)	3.53 ± 0.83 3.57 (2.06–4.83)	3.76 ± 1.3 3.44 (1.6–7.1)
Sq(µm)	T1	3 ± 0.78 2.93 (0.97–4.21)	4.03 ± 0.92 4.08 (1.92–5.3)	4.83 ± 0.79 4.98 (3.04–5.96)	5.73 ± 1.62 5.57 (3.02–8.89)
	T2	2.71 ± 1.09 2.48 (0.87–5.36)	3.36 ± 0.93 3.14 (1.67–4.92)	3.80 ± 0.86 3.78 (2.03–5.77)	4.21 ± 0.96 4.11 (2.23–6.2)

Min.=Minimum; Max.=Maximum; SD = standard deviation

**Table 5:** Evaluation of the time-dependent changes in enamel surface roughness values within the groups

		Group 1 A		Group 1B		Group 2 A		Group 2B	
Variables	Time	Mean ± SD Median (Min.-Max.)	*p*- in-group	Mean ± SD Median (Min.-Max.)	*p*- in-group	Mean ± SD Median (Min.-Max.)	*p*- in-group	Mean ± SD Median (Min.-Max.)	*p*- in-group
Ra(µm)	T1-T0	0.04 ± 0.05 0.03 (-0.04-0.14)	***0.003*** ^***a***^ *	0.1 ± 0.09 0.11 (-0.1-0.25)	***< 0.001*** ^a*^	0.07 ± 0.06 0.05 (-0.01-0.18)	***< 0.001*** ^b*^	0.13 ± 0.1 0.10 (0.02–0.38)	***< 0.001*** ^b*^
	T2-T1	-0.11 ± 0.1 -0.1 (-0.37-0.01)	***< 0.001*** ^***b***^ *	-0.09 ± 0.07 -0.07 (-0.25-0.0)	***< 0.001*** ^a*^	-0.08 ± 0.07 -0.08 (-0.22-0.04)	***< 0.001*** ^a*^	-0.13 ± 0.11 -0.11 (-0.41-0.04	***< 0.001*** ^a*^
	T2-T0	-0.07 ± 0.12 -0.04(-0.39-0.12)	***0.010*** ^***a***^ *	0.01 ± 0.06 0.00 (-0.16-0.12)	0.286^a^	-0.01 ± 0.07 0.00 (-0.19-0.17)	0.477^a^	0.00 ± 0.07 0.01 (-0.17-0.15)	0.897^a^
Rq(µm)	T1-T0	0.03 ± 0.12 0.08 (-0.23-0.2)	0.209^a^	0.1 ± 0.09 0.09 (-0.02-0.31)	***< 0.001*** ^a*^	0.07 ± 0.09 0.04 (-0.08-0.23)	***0.001*** ^a*^	0.10 ± 0.09 0.09 (-0.07-0.3)	***< 0.001*** ^a*^
	T2-T1	-0.07 ± 0.13 -0.08 (-0.46-0.13)	***0.012*** ^***a***^ *	-0.11 ± 0.1 -0.1 (-0.34-0.13)	***< 0.001*** ^a*^	-0.09 ± 0.1 -0.08 (-0.27-0.07)	***< 0.001*** ^a*^	-0.09 ± 0.08 -0.09(-0.25-0.05)	***< 0.001*** ^a*^
	T2-T0	-0.04 ± 0.13 -0.03 (-0.35-0.18)	0.159^a^	-0.01 ± 0.11 -0.02(-0.22-0.34)	0.810^a^	-0.01 ± 0.1 0.0 (-0.25-0.21)	0.509^a^	0.01 ± 0.1 0.02 (-0.31-0.15)	0.236^b^
	T1-T0	0.79 ± 0.48 0.82 (-0.26-1.63)	***< 0.001*** ^***a***^ *	1.45 ± 0.69 1,47 (0.27–3.32)	***< 0.001*** ^a*^	1.28 ± 1.09 1.39 (-0.68-2.78)	***< 0.001*** ^a*^	2.01 ± 1.01 2.08 (-0.16-3.92)	***< 0.001*** ^a*^
Sa(µm)	T2-T1	-0.04 ± 0.66 -0.23 (-0.91-1.20)	0.548^b^	-0.18 ± 0.89 -0.23(-1.45-1.97)	0.357^a^	-0.71 ± 1.15 -0.50 (-3.81-0.91)	***0.008*** ^a*^	-1.18 ± 1.21 -1.09(-3.82-1.52)	***0.001*** ^b*^
	T2-T0	0.76 ± 0.64 0.69 (-0.30-1.86)	***< 0.001*** ^***a***^ *	1.27 ± 1.01 1.13 (-1.15-3.01)	***< 0.001*** ^a*^	0.57 ± 1.09 0.38 (-1.70-2.29)	0.023^a^	0.82 ± 0.95 0.85 (-1.35-2.92)	***0.001*** ^a*^
	T1-T0	1.18 ± 0.95 1.20 (-1.35-2.62)	***< 0.001*** ^***a***^ *	1.60 ± 1.11 1.59 (-1.17-3.87)	***< 0.001*** ^a*^	1.30 ± 1 1.22 (-0.14-3.21)	***< 0.001*** ^a*^	1.96 ± 1.13 2,01 (-0.4-4)	***< 0.001*** ^a*^
Sq (µm)	T2-T1	-0.29 ± 0.79 -0.34 (-1.67-2.29)	***0.012*** ^***b***^ *	-0.67 ± 1.03 -0.39 (-2.8-1.08)	***0.006*** ^a*^	-1.04 ± 1.04 -1.00 (-2.81-0.88)	***< 0.001*** ^a*^	-1.52 ± 1.15 -1.2.(-4.08-0.31)	***< 0.001*** ^a*^
	T2-T0	0.89 ± 0.84 0.66 (-0.3-3.12)	***< 0.001*** ^***a***^ *	0.93 ± 1.1 0.90 (-1.22-3.41)	***0.001*** ^a*^	0.27 ± 0.78 0.21 (-1.7-2.04)	0.128^a^	0.44 ± 1.27 0.90 (-2.45-2.22)	0.077^b^

*p < 0,017 was significant

p^a^: Paired Sample T-Test was used

p^b^: Wilcoxon Test was used

Min.=Minimum; Max.=Maximum; SD = Standard deviation

According to the inter-group comparison results, the differences between the parameters Ra (T1-T0), Ra (T2-T0), Rq (T1-T0), and Sa (T1-T0) were statistically significant when comparing groups 1 A and 1B for the flowable composite treated samples. These changes were significantly greater in the group using tungsten carbide burs (1B) compared to the group using fiberglass burs (1 A) (*p* < 0.05) (Table 6).

The differences between the parameters Ra (T1-T0), Sa (T1-T0), and Sq (T1-T0) were determined to be statistically significant when comparing Groups 2 A and 2B for the samples treated with packable composite. The changes observed in the group with tungsten carbide burs (2B) were significantly greater than those in the group with fiberglass burs (2 A) (Table 6).

The impact of composite type on roughness changes was analyzed for the differences between T1-T0 and T2-T0 times. The differences in Ra (T2-T0) and Sq (T2-T0) parameters between the different composite resin groups (Group 1 A and 2 A) for specimens with fiber-supported composite bur application after using attachment debonding pliers (Group A) were statistically significant (*p* < 0.05). When analyzing the Sq (T2-T0) parameter, an increase in roughness values was observed after polishing compared to the initial roughness value in both groups. Additionally, the difference between the polishing process and the initial roughness value was greater in Group 1 A than in Group 2 A (Table 6).

The difference in the Sa (T1-T0) parameters between the composite resin groups (Group 1B and 2B) for the specimens subjected to carbide bur-renew stone application (Group B) was statistically significant (*p* < 0.05). There was a larger increase in the Sa (T1-T0) parameter roughness in Group 2B compared to Group 1B (Table 6).

**Table 6:** Comparison of time-dependent changes in enamel surface roughness values between the groups

Change in Parameters Over Time	Groups 1 A-1B Mean Difference (95%Confidence Interval)	Groups 2 A-2B Mean Difference (95%Confidence Interval)	Groups 1–2 A Mean Difference (95%Confidence Interval)		Groups 1B-2B Mean Difference (95%Confidence Interval)	*p*- between groups
Ra T1-T0 (µm)	0.069 (0.023–0.115)	0.067 (0.021–0.113)	0.031 (-0.015-0.077)		0.029 (-0.017-0.075)	0.004* (1B > 1 A) 0.005* (2B > 2 A) 0.188 (1–2 A) 0.211 (1B-2B)
Rq T1-T0 (µm)	0.068 (0.009–0.128)	0.028 (-0.031-0.088)	0.041 (-0.019-0.101)		0.001 (-0.059-0.061)	0.026* (1B > 1 A) 0.349 (2 A-2B) 0.175 (1–2 A) 0.971 (1B-2B)
Sa T1-T0 (µm)	0.653 (0.140– 1.167)	0.067 (0.021–0.113)	0.484 (-0.029– 0.998)		0.559 (0.046– 1.073)	0.013* (1B > 1 A) 0.005* (2B > 2 A) 0.064 (1–2 A) 0.033* (2B > 1B)
Sq T1-T0 (µm)	0.426 (-0.204-1.055)	0.664 (0.034–1.293)	0.125 (-0.504-0.755)		0.363 (-0.267-0.992)	0.182(1 A-1B) 0.039* (2B > 2 A) 0.363 (1–2 A) 0.255 (1B-2B)
Ra T2-T0 (µm)	0.090 (0.038–0.142)	0.013 (-0.038-0.065)	0.063 (0.012–0.115)		0.013 (-0.039-0.065)	0.001* (1B > 1 A) 0.609. (2 A-2B) 0.017* (2 A > 1 A) 0.620 (1B-2B)
Rq T2-T0 (µm)	0.033 (-0.033-0.100)	0.024 (-0.042-0.090)	0.025 (-0.041-0.091)	0.015 (-0.051-0.081)		0.317 (1 A-1B) 0.477 (2 A-2B) 0.458 (1–2 A) 0.651 (1B-2B)
Sa T2-T0 (µm)	0.512 (-0.050-1.074)	0.258 (-0.304-0.820)	0.188 (-0.374-0.750)	0.443 (-0.119-1.004)		0.074 (1 A-1B) 0.364 (2 A-2B) 0.507 (1–2 A) 0.121 (1B-2B)
Sq T2-T0 (µm)	0.045 (-0.566-0.656)	0.178 (-0.432-0.789)	0.620 (0.010–1.231)	0.487 (-0.123-1.098)		0.884 (1 A-1B) 0.563 (2 A-2B) 0.047* (1 A > 2 A) 0.116 (1B-2B)
Ra T2-T1 (µm)	0.021 (-0.034-0.075)	0.054 (-0.001-0.109)	-	-		0.452 (1 A-1B) 0.052 (2 A-2B)
Rq T2-T1 (µm)	0.035 (-0.027-0.097)	0.004 (-0.058-0.067)	-	-		0.270 (1 A-1B) 0.877 (2 A-2B)
Sa T2-T1 (µm)	0.141 (-0.460-0.742)	0.470 (-0.130-1.071)	-	-		0.642 (1 A-1B) 0.123 (2 A-2B)
Sq T2-T1 (µm)	0.381 (-0.226-0.988)	0.485 (-0.122-1.092)	-	-		0.216 (1 A-1B) 0.116 (2 A-2B)

*p < 0.05 was significant

The effects of the two factors of interest (Factor 1 (composite type): Group 1 or 2; Factor 2 (technique): Group A or B) on the changes between times were evaluated using the results of the Bonferroni pairwise comparison test, which were obtained from a two-way analysis of variance (two-way ANOVA)

“-”: Not Compared (The impact of composite type on roughness changes was not analyzed for the differences between T2-T1 times)

## Discussion

This study examined the effects of two different evidence-based methods for removing residual composite in orthodontics on the enamel surface after debonding, focusing on the roughness of the enamel surface and the topographic changes that occur during the removal process. This study’s results showed significant differences in the surface roughness and topographic changes of the enamel between the two debonding and polishing methods. Therefore, the primary tested null hypothesis was rejected. The results from the quantitative evaluations using Geomagic Control X software in this study indicate that the amount of composite removed with the pliers was not influenced by the viscosity of the composite. As a result, the second null hypothesis tested was accepted.

Attachments used in clear aligner treatments are applied to the tooth surface to enhance retention and facilitate challenging tooth movements while maintaining the aesthetic appearance of the treatment [22]. During the final stage of orthodontic treatment with aligners, the bonded composite attachments must be removed from the tooth surface [23]. Removing superficial enamel after debonding may reduce its resistance to organic acids in the oral environment, potentially increasing the risk of demineralization [24]. This study aimed to evaluate the enamel surface roughness after removing attachments formed with flowable and packable composites with two different fillers and viscosities from the tooth surface using different removal methods (fiberglass-tungsten carbide bur) using a 3D non-contact optical profilometer. Additionally, we aimed to assess the morphological changes on the enamel surface in the groups treated with packable composite resin using 3D micro-CT.

During the debonding phase of aligner treatments, more airborne particles are inevitably generated compared to those produced during the debonding phase of conventional fixed treatments. As Eliades et al. [13] noted in their study, due to the harmful effects of the particles produced during composite removal, we opted to use an attachment debonding plier in one subgroup for each composite resin group for debonding. To the best of our knowledge, no study has assessed the amount of composite remaining on the tooth surface of composite attachments detached using an attachment debonding plier. According to the results of quantitative evaluations using Geomagic Control X software in our study, over 50% of the total attachment volume was removed with the plier for both composite types, and the viscosity of the composite did not affect the results. The ability to remove most attachments with an attachment debonding plier is a crucial step in safeguarding the health of both the patient and the clinician, and it can be considered to be routinely added to the debonding stage of aligner attachments.

Additionally, when selecting the composite for attachment construction, we believe that attachment debonding pliers exert the same effect on the removal of both flowable and packable composite types, clinically. Both composite types can be chosen based on the clinician’s preference.

While some clinicians prefer flowable composites with low filler amounts due to their short application time and ease of use, others favor high-viscosity composites with high filler amounts for attachment, as they may provide a greater stability advantage [3, 4, 25]. In the present study, the roughness measurements taken after applying the tungsten carbide bur method (Group 1B-Group 2B) in the debonding process of attachments made with flowable composite (Group 1) and packable composite (Group 2) revealed a greater increase in roughness in Group 2B compared to the initial condition when examining the Sa (T1-T0) parameter. Due to the high filler particle content in packable composites, their viscosity and durability are increased [26]. Thus, during the debonding process of attachments made with packable composites, increased force may be applied to the tooth surface, and more contact may be necessary when removing residual composite resins. This result suggests that attachments made with packable composites may lead to an increase in roughness after the debonding stage. However, additional studies on this subject are necessary.

Ruiz et al. [23], Karan et al. [11], Almudhi et al. [27], and Thawaba et al. [17] found that zirconia burs resulted in the lowest enamel surface roughness and the least enamel damage when compared to tungsten carbide burs. Similarly, this study found that when comparing tungsten carbide bur combined with renew stone, the groups treated with the fiberglass method exhibited a smoother enamel surface after debonding the attachment with pliers, as measured by a 3D optical profilometer. No significant difference was observed between the methods applied at the polishing stage (change from T1 to T2), and both methods reduced the surface roughness after polishing. This indicates that the method selected during the debonding stage is more significant than the one chosen in the polishing process, and it suggests that the polishing step should be incorporated into the finishing procedure. Similar to our results, Shah et al. [6] also emphasized in their study that the roughness values after polishing in all debonding methods approached the initial values but still remained higher. These results highlight that the enamel surface cannot be fully restored, as demonstrated in our study. We preferred to use white fiberglass with blue fiberglass, which is indicated for polishing after debonding. Since fiberglass burs work slower when compared to tungsten burs, we preferred to remove the attachments with adhesive removing pliers in this group. As in our study, the Renew stone can be applied for polishing after removing excess composite with a carbide bur or used directly to clean residual composite, as shown by Almudhi et al. [27].

In their recent in vitro study, Thys et al. [28] suggested that systems with more polishing steps could cause greater abrasion on the tooth surface, thereby reducing roughness. Based on their findings, we incorporated the Micro-CT measurement method into our study to investigate whether there is increased wear on the enamel surface due to smoother enamel being formed after debonding. We conducted these measurements in the packable composite group (Group 2), where we anticipated observing more roughness.

In the study by Cesur et al. [16], which is the only research in the literature examining enamel structure after debonding using the Micro-CT method, the application of pumice with an 8-blade carbide bur, Detartrine paste with a fiber-supported stainbuster composite bur, and Sof-Lex discs from the thickest to the thinnest in the subgroups were compared. According to the study results, the greatest damage was caused in the group where pumice was applied with tungsten carbide bur. In contrast to the findings of Cesur et al. [16], the present study revealed that the application of fiberglass following attachment debonding with pliers resulted in a more significant loss in both the demineralization area and volume change when compared to the tungsten carbide bur group. This difference in our study may have arisen from using tungsten carbide burs with more blades, unlike the study by Cesur et al. [16], which used burs with fewer blades. There may have been invisible enamel damage during the removal of the attachments with the pliers, which may have caused more enamel loss than expected in the fiberglass group. In the systematic review by Paolone et al. [24], the volumetric loss of enamel ranged from 0.02 ± 0.01 mm³ to 0.61 ± 0.51 mm³ per tooth. When we examine the micro-CT results of our study, it is evident that the level of demineralization occurring in the two groups is acceptable. In the study by Suliman et al. [29], it was stated that the depth of demineralization is a more clinically important measurement. This study found no significant difference in demineralization depth between Group 2 A and Group 2B.

When we examine our results, the changes in the Ra (T1-T0), Sa (T1-T0), and Sq (T1-T0) roughness parameters were significantly less in Group 2 A compared to Group 2B. On the other hand, micro-CT results indicated greater changes in demineralization volume and area in Group 2 A compared to those observed in Group 2B. Therefore, while the fiberglass method created a smoother enamel surface, it also resulted in more enamel loss. Since there are few studies on this subject that utilize 3D analysis with micro-CT, which is the gold standard for evaluating the mineralization of hard tissues, more future studies need to be conducted to reach a clear conclusion on this matter.

Since intraoral factors can influence the bond strength of the composite, they may also alter the enamel damage that occurs after debonding [29]. The limitation of the present study is that it is an in-vitro study, and oral factors such as saliva, temperature, and pH were not considered; therefore, the results should be interpreted more cautiously. On the other hand, studying advanced enamel surface evaluation technology in sufficient sample groups is a very important step to obtain meaningful results.

## Conclusions

Based on the findings of this in-vitro study, the following conclusions were reached:

1. Over 50% of the total attachment volume was removed with the plier for both composite types, and the results were not influenced by the composite’s viscosity.
2. Fiberglass used with a debonding plier resulted in a smoother enamel surface compared to carbide burs, but it caused significantly greater enamel demineralization, as demonstrated by micro-CT evaluations after polishing.

## Data Availability

No datasets were generated or analysed during the current study.
